# Supplementary material for: Bile salt hydrolase catalyses formation of amine-conjugated bile acids
Source: Nature. 2024 Feb 7;626(8000):859–63. doi: 10.1038/s41586-023-06990-w (PMC10881385; doi:10.1038/s41586-023-06990-w)
Supplement: Supplementary file 1 — Reporting Summary [file 41586_2023_6990_MOESM1_ESM.pdf]

nature portfolio

Corresponding author(s): Andrew D. Patterson

Last updated by author(s): 11/17/2023

Reporting Summary

Nature Portfolio wishes to improve the reproducibility of the work that we publish. This form provides structure for consistency and transparency in reporting. For further information on Nature Portfolio policies, see our Editorial Policies and the Editorial Policy Checklist.

Statistics

For all statistical analyses, confirm that the following items are present in the figure legend, table legend, main text, or Methods section.

n/a	Confirmed	
☐	☒	The exact sample size (*n*) for each experimental group/condition, given as a discrete number and unit of measurement
☐	☒	A statement on whether measurements were taken from distinct samples or whether the same sample was measured repeatedly
☐	☒	The statistical test(s) used AND whether they are one- or two-sided<br>*Only common tests should be described solely by name; describe more complex techniques in the Methods section.*
☐	☒	A description of all covariates tested
☐	☒	A description of any assumptions or corrections, such as tests of normality and adjustment for multiple comparisons
☐	☒	A full description of the statistical parameters including central tendency (e.g. means) or other basic estimates (e.g. regression coefficient) AND variation (e.g. standard deviation) or associated estimates of uncertainty (e.g. confidence intervals)
☐	☒	For null hypothesis testing, the test statistic (e.g. *F*, *t*, *r*) with confidence intervals, effect sizes, degrees of freedom and *P* value noted<br>*Give P values as exact values whenever suitable.*
☒	☐	For Bayesian analysis, information on the choice of priors and Markov chain Monte Carlo settings
☐	☒	For hierarchical and complex designs, identification of the appropriate level for tests and full reporting of outcomes
☐	☒	Estimates of effect sizes (e.g. Cohen's *d*, Pearson's *r*), indicating how they were calculated

Our web collection on statistics for biologists contains articles on many of the points above.

Software and code

Policy information about availability of computer code

Data collection	Bruker TopSpin - ver. 4.0.9<br>Waters MassLynx - ver. 4.1<br>Thermo Xcalibur (TSQ Quantis Plus) - ver. 4.5.474.0<br>Thermo Xcalibur (Orbitrap Exploris 120) - ver. 4.6.67.17
Data analysis	GraphPad Prism - ver. 6.07<br><br>Chenomx NMR Suite - ver. 9.02<br>Waters TargetLynx - ver. 4.1<br>Thermo Xcalibur (TSQ Quantis Plus) - ver. 4.5.474.0<br>Thermo Xcalibur (Orbitrap Exploris 120) - ver. 4.6.67.17<br>MS-DIAL- ver. 4.9.221218<br>R - ver. 4.2.2<br><br>The code used for analysis and generating the figures are found at GitHub (https://github.com/PattersonLab-PSU/BBAAs-paper) and Zenodo (10.5281/zenodo.10072966).

For manuscripts utilizing custom algorithms or software that are central to the research but not yet described in published literature, software must be made available to editors and reviewers. We strongly encourage code deposition in a community repository (e.g. GitHub). See the Nature Portfolio guidelines for submitting code & software for further information.

Data

Policy information about availability of data

All manuscripts must include a data availability statement. This statement should provide the following information, where applicable:

- Accession codes, unique identifiers, or web links for publicly available datasets
- A description of any restrictions on data availability
- For clinical datasets or third party data, please ensure that the statement adheres to our policy

RNA-sequencing datasets are deposited in NCBI SRA Database under bioproject: PRJNA878764. BBAA quantification data (Figure 1) were obtained from earlier published studies: doi.org/10.21203/rs.3.rs-820302/v1 and 10.1128/mSystems.00805-21 and corresponding bacterial genomes for comparative genomics were obtained from NCBI RefSeq (see Figure 1D source data and GitHub). For the human study, raw triple quadrupole mass spectrometry data have been deposited in the Mass Spectrometry Interactive Virtual Environment (MassIVE) under accession number MSV000093144. Structures of Bifidobacterium longum and Clostridium perfringens BSH were acquired from the Protein Database (PDB: 2HF0 and 2BJF, respectively). The Infant Growth and Microbiome (IGram) Study enrolled pregnant African American mothers and their newborn infants. The purpose of the research study was to learn more about the bacteria normally living in the child's gut, how it is transferred from mother to child, and whether it affects the child's growth in the first three years of life. The IGram data used in this publication were consistent with the stated purpose of the research. The IRB approved consent documents included language that allowed participants to indicate if they would like to have their information included in future research. Subjects may participate in the original research without their information (even if de-identified) being included in future research. Therefore, the data submitted to the repository (SRA accessions PRJNA1042647 and PRJNA557731) were limited to those individuals who consented to future use of their data and are not the entire data set used in the analyses presented here. To request the complete data set, contact Babette Zemel, PhD (zemel@chop.edu) or Kyle Bittinger, PhD (bittingerk@chop.edu), with a summary of how the data will be used and how it is consistent with the goals of the study. The request will be reviewed by the study team at the Children's Hospital of Philadelphia. If approved, the data will be made available within one month at no cost.

Human research participants

Policy information about studies involving human research participants and Sex and Gender in Research.

Reporting on sex and gender	56 male and 52 female subjects were used for the human study.
Population characteristics	Pregnant African American women in their third trimester who had a pre-pregnancy BMI in the healthy or obese range and were enrolled, provided they were carrying singletons, and were free of medical conditions associated with glucose regulation, immunosuppresants, chronic inflammatory, or autoimmune diseases. Their infants were enrolled at birth if they were term, and free of chromosomal anomalies and conditions affecting growth and development.
Recruitment	Pregnant women receiving care in the obstetrics clinics at the Hospital of the University of Pennsylvania and who met the enrollment criteria were invited to participate.
Ethics oversight	The study protocol was reviewed and approved by the Committee for the Protection of Human Subjects (Internal Review Board) of the Children's Hospital of Philadelphia.

Note that full information on the approval of the study protocol must also be provided in the manuscript.

Field-specific reporting

Please select the one below that is the best fit for your research. If you are not sure, read the appropriate sections before making your selection.

☒ Life sciences ☐ Behavioural & social sciences ☐ Ecological, evolutionary & environmental sciences

For a reference copy of the document with all sections, see nature.com/documents/nr-reporting-summary-flat.pdf

Life sciences study design

All studies must disclose on these points even when the disclosure is negative.

Sample size	The metagenomics and bile acid analysis of the human study were secondary and exploratory so no power calculation was performed to determine sample size. The gnotobiotic experiment sample size was determined based on access to germ free mice due to housing and breading limitations.
Data exclusions	No data was excluded.
Replication	All experiments were successfully repeated at least two times. The human study was not repeated.
Randomization	Bile acid quantification samples were randomized prior to LC-MS/MS analysis. The human study was prospective so there was no subject randomization. Gnotobiotic mice were randomly assigned to the two treatment groups.

Blinding	Blinding was not part of the study protocol and was not used.

Reporting for specific materials, systems and methods

We require information from authors about some types of materials, experimental systems and methods used in many studies. Here, indicate whether each material, system or method listed is relevant to your study. If you are not sure if a list item applies to your research, read the appropriate section before selecting a response.

Materials & experimental systems

n/a	Involved in the study
☒	☐ Antibodies
☐	☒ Eukaryotic cell lines
☒	☐ Palaeontology and archaeology
☐	☒ Animals and other organisms
☒	☐ Clinical data
☒	☐ Dual use research of concern

Methods

n/a	Involved in the study
☒	☐ ChIP-seq
☒	☐ Flow cytometry
☒	☐ MRI-based neuroimaging

Eukaryotic cell lines

Policy information about cell lines and Sex and Gender in Research

Cell line source(s)	Cell lines for the luciferase-based reporter assays were obtained from INDIGO Biosciences.
Authentication	Authentication was performed by INDIGO Biosciences.
Mycoplasma contamination	Cell lines are free of mycoplasma based on INDIGO Biosciences' report.
Commonly misidentified lines (See ICLAC register)	N/A

Animals and other research organisms

Policy information about studies involving animals; ARRIVE guidelines recommended for reporting animal research, and Sex and Gender in Research

Laboratory animals	Male 8- to 10-week-old C57BL/6J mice were maintained under germ-free conditions in positive pressure isolators.
Wild animals	N/A
Reporting on sex	15 male mice were used in the B. fragilis NCTC 9343 or the Δbsh mutant monocolonization experiments.
Field-collected samples	N/A
Ethics oversight	Gnotobiotic experiments were conducted under the Pennsylvania State University Institutional Animal Care and Use Committee approved protocol 202101826

Note that full information on the approval of the study protocol must also be provided in the manuscript.
